# Implementation of patient-reported outcomes in personalized lung cancer care: a qualitative study from the perspective of patients and health care providers

**DOI:** 10.1186/s41687-026-01091-4

**Published:** 2026-05-28

**Authors:** Vanessa Mildenberger, Dusan Simic, Leonie Eilers, Anika Kästner, Marie Naumann, Anna Rasokat, Anna Kron, Daniel Armbrust, Anna Spier, Neeltje van den Berg, Wolfgang Hoffmann, Jürgen Wolf, Florian Kron, Stephanie Stock

**Affiliations:** 1 Faculty of Medicine and University Hospital Cologne, Institute for Health Economics and Clinical Epidemiology, University of Cologne, Cologne, Germany; 2 KCM KompetenzCentrum für Medizinoekonomie, FOM Hochschule für Oekonomie & Management gemeinnützige Gesellschaft mbH, Essen, Germany; 3 Institute for Community Medicine, Section Epidemiology of Health Care and Community Health, University Medicine Greifswald, Greifswald, Germany; 4 Faculty of Medicine, Department of Internal Medicine, Center for Integrated Oncology (CIO) Aachen Bonn Cologne, University of Cologne, University Hospital Cologne, Cologne, Germany; 5 Faculty of Medicine and University Hospital Cologne, University of Cologne, National Network Genomic Medicine (nNGM) Lung Cancer, Cologne, Germany

**Keywords:** Patient-reported outcomes, Patient-centered care, Lung cancer, Non-small-cell lung cancer, Personalized lung cancer therapy, Implementation, Formative evaluation, Qualitative interviews

## Abstract

**Background:**

Patient-Reported Outcomes (PROs) in personalized lung cancer care aim to facilitate a targeted therapeutic management approach. This study explores the implementation of PROs as an intervention component within a precision medicine program, with a focus on identifying facilitating factors and barriers from the perspectives of health care providers (HCPs) and patients with stage IV non-small-cell lung cancer (NSCLC).

**Methodology:**

Semi-structured individual interviews were conducted with HCPs and NSCLC patients over two rounds in 2023 and 2024, respectively. Verbatim transcripts were analyzed using qualitative content analysis. The framework of Grol and Wensing was employed, which divides healthcare into six dimensions.

**Results:**

The structured documentation of symptoms and quality of life constitutes a benefit in that it enables intervention by the treating physician. From the perspective of HCPs, incorporating PROs into daily clinical practice was associated with considerable organizational challenges and the need for logins into an additional system to access the results. This required a substantial investment of time and personnel, which resulted in a lack of acceptance. The predominant motivation of patients who participated in the PRO questionnaires was a desire to contribute to scientific research. In general, the regular administration of the questionnaire has proven unfeasible for the NSCLC cohort, given the high disease burden often experienced in stage IV.

**Conclusions:**

Despite implementation challenges, PROs can improve symptom monitoring, communication, and patient-centered care in advanced NSCLC when key barriers are addressed.

## Background

In recent years, patient-centered care (PCC) has become increasingly relevant in the context of healthcare delivery reforms. Driven by both medical innovation such as precision medicine in cancer treatment and the reform pressure to adapt healthcare to the increasing prevalence of chronic diseases, a variety of medical disciplines have adopted PCC [1]. In lung cancer care, these aspects are of considerable importance, as the administration of targeted drugs can prolong survival by several years while causing fewer adverse events compared to chemotherapy alone [2, 3]. Lung cancer is the leading cause of cancer-related mortality worldwide and in Germany [4, 5]. In addition to the medical aspect, a holistic, patient-centered approach is to be developed to assess every aspect of the patient’s condition [6, 7]. Accordingly, in the context of oncology, the consideration of the overall health status extends beyond biological and medical aspects to include the psychological state and health-related quality of life (HRQoL) [6, 8, 9].

Meanwhile, Patient-Reported Outcomes (PROs) have emerged as the prevailing standard for assessing HRQoL from the patient perspective. This approach may also contribute to healthcare providers monitoring cancer-related symptoms [10, 11]. A systematic review with meta-analysis reveals that the application of PROs in cancer care may prolong overall survival and improve quality of life [12]. In Germany, their utilization has predominantly been demonstrated to be advantageous in the context of clinical and epidemiological research projects [13, 14]. PROs are particularly crucial in lung cancer, where patients often suffer from disease-related symptoms or treatment-related toxicities that affect their daily lives [15, 16]. Lung-cancer–specific immunotherapy PROM programs and the ongoing PRO-NET randomized trial have adapted their symptom libraries and PRO item sets to capture immune-related toxicities [17, 18].

The systematic documentation of PROs constitutes an intervention component in the DigiNet project (NCT05818449), which is funded by the Innovation Fund of the Federal Joint Committee (Gemeinsamer Bundesausschuss, GBA) in Germany (01NVF20021) [19]. DigiNet builds on the National Network Genomic Medicine Lung Cancer (nNGM), which delivers molecular diagnostics and personalized therapy guidance after the initial diagnosis (NCT05934032) [20]. The objective of DigiNet was to establish and enhance digital networking in personalized lung cancer therapy for patients with stage IV non-small-cell lung cancer (NSCLC) in Germany, focusing on three model regions: the federal states of North Rhine-Westphalia, Berlin and Saxony. Over the course of the study, PROs were assessed at four-week intervals by patients via the patient portal or paper-based. The standardized and validated questionnaires cover the areas of HRQoL, symptom control, functional status, mobility, anxiety and depression and can be completed by patients via the shared database or paper-based. Patients have the option of viewing their completed questionnaires at any time. The following questionnaires are implemented [21–23]:

- European Organization For Research And Treatment Of Cancer (EORTC) Quality of Life Questionnaire Core Module (QLQ-C30) – questionnaire for assessing the quality of life of cancer patients.
- EORTC lung cancer module (QLQ-LC29) – questionnaire for assessing the quality of life of lung cancer patients.
- Patient Health Questionnaire-4 (PHQ-4) – ultra-short screening for anxiety and depression.

PRO results and changes over time were visualized in real-time for the treating physician via the central data platform. Paper-based questionnaires must first be entered into the database by assistants, thereby enabling physicians to access the results. The DigiNet intervention involves the physician in monitoring this information during the follow-up visits and incorporating the results into their clinical decision-making.

Despite the potential for enhancing the treatment of patients with lung cancer, the application of PROs in routine medical care comes with certain challenges, including the disruption of clinical workflows [24]. There is a lack of knowledge surrounding the optimal implementation process, and there is a need to generate an understanding of facilitating and hindering factors, as well as the mechanisms through which these factors take effect [24, 25]. Previous studies were often conducted in other countries where healthcare sectors may differ [26–28], or focused on the proof of effectiveness of clinical endpoints, adopting the design of a randomized controlled study [10, 15, 29]. The objective of this study is to generate insights into the implementation of PROs as an intervention component within an established precision oncology program, with a focus on identifying the facilitating factors and barriers from the perspectives of HCPs and patients with advanced NSCLC.

## Methods

### Study design and participants

A qualitative study design was chosen for this study. This enables an in-depth exploration of yet unexplored empirical phenomena [30]. The objective of conducting semi-structured interviews is to obtain a comprehensive range of opinions from key stakeholders who have been directly involved in the implementation process. The active involvement of both HCPs and patients is a prerequisite for successful implementation. Consequently, a comprehensive analysis is necessary to consider the perspectives of both parties equally. The study is reported in accordance with the COREQ checklist [31].

**Fig. 1:**
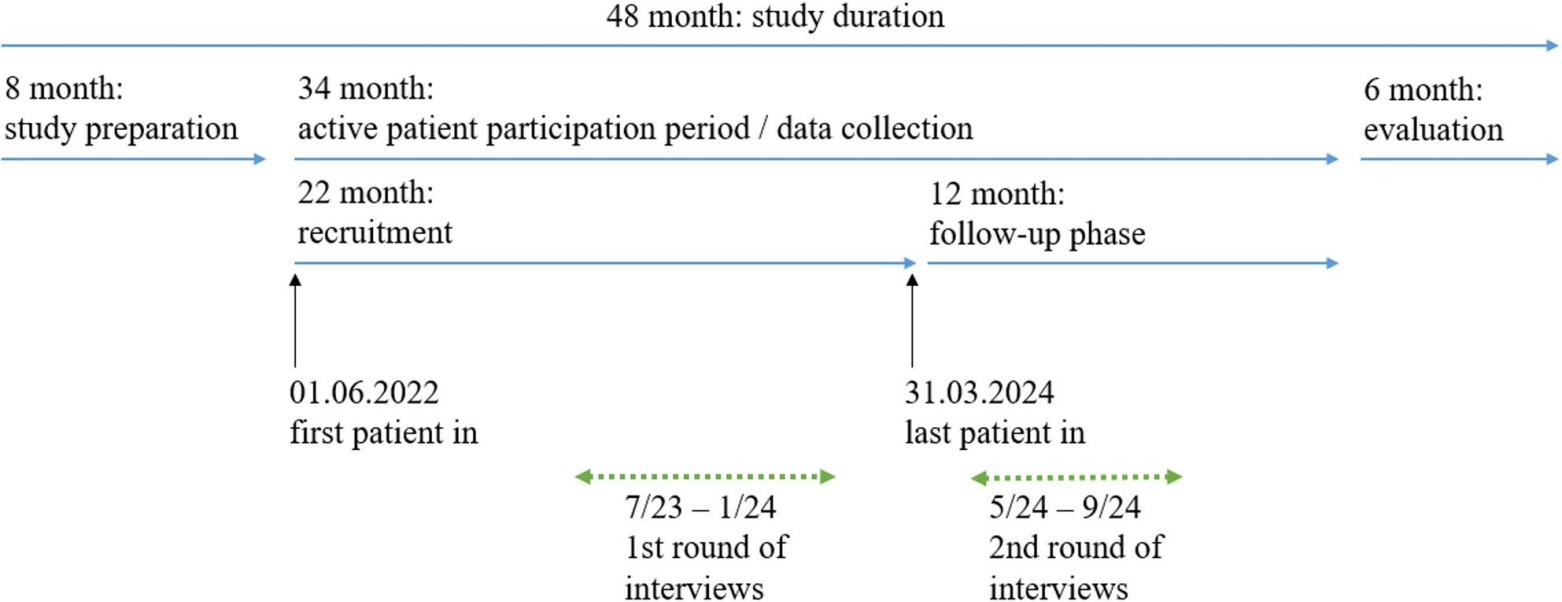
Overview of DigiNet study periods

This qualitative study was conducted as part of the formative evaluation of the prospective, controlled, non-randomized, multicenter cohort study DigiNet, with the primary aim of evaluating whether the optimized digital networking and enhancement of precision oncology improves survival and HRQoL of patients diagnosed with NSCLC. The study period lasted four years from September 1, 2021, to September 30, 2025, and was divided into the following phases, which are illustrated in Fig. 1: study preparation (eight months), recruitment of the DigiNet intervention group (22 months), follow-up phase of the intervention group (12 months), and evaluation (six months). The active study phase is now concluded and the evaluation phase is ongoing.

According to the study protocol, it was intended that two rounds of interviews should be conducted with approximately 20 HCPs and 20 patients. The HCP cohort included physicians, study coordinators, data managers and study assistants. For data protection reasons, recruitment of interview partners was conducted in collaboration with the trust office. All the HCPs involved in the study were invited via e-mail to participate in the interview. In the first round, patients were initially invited to the interview by sending a letter using a random sample. Due to an initially low response rate, patients who had registered on the patient portal were additionally invited by e-mail via the portal. In the second round, a randomly selected sample was invited by sending a letter to recruit patients who only participate in the paper-based format. To obtain a purposive sample, filters were applied based on factors such as study region (federal state and rural vs. urban), age, and PRO participation (digital vs. paper-based). No financial incentives were provided for participation in the interview.

### Data collection

Semi-structured individual interviews were conducted in two phases: the first from June 2023 to January 2024, and the second from May 2024 to September 2024. In cases of time constraints of the HCPs, a joint interview was arranged when participants were located at the same clinical site, for example, interviews involving both a clinician and their study nurse. If a patient’s health condition did not allow for an individual interview, participation together with a designated support person, such as their spouse, was permitted.

No relationship existed between the interviewer and the participants. All the interviews were conducted by the first author, VM, who was a research associate at the University of Cologne at the Institute of Health Economics and Clinical Epidemiology with experience in the field of qualitative research.

An interview guide was used, which was developed based on a systematic literature review. It consisted of several thematic sections with guiding questions, optional follow-up questions and narrative impulses to allow for the use of an open-ended interview design, depending on how the conversations unfolded [32]. The entire interview guide was developed in accordance with the superordinate research question for the formative evaluation.

The specific thematic section that focused on PROs as an intervention component was part of the questionnaire. For HCPs, the fundamental question of this section pertained to their experiences thus far with the PROs at their clinical site. Subsequent inquiries addressed patient participation, format, PRO organization, and the perceived benefits. The patients were firstly asked whether they completed PRO questionnaires regularly. If not, the reasons for this were explored. If they had participated in at least one PRO survey, the questions referred to their emotional response to the questionnaire, perceived benefit, format preference (digital vs. paper-based), and suggestions for improvement. The interview guides for providers and patients respectively were pre-tested and subsequently slightly adapted.

The sample size for the formative evaluation was based on data saturation and was defined as adequate when there was sufficient data material to answer the research questions, a large part of the material was repetitive in content, and a range of opinions was included. HCPs and patients were permitted to opt for either a digital interview conducted via Zoom or a telephone interview, according to their personal preference. Prior to their participation, all respondents were informed of the objective of the interview in the context of the DigiNet project and data management and given the opportunity to ask further questions.

### Data analysis

Verbatim transcripts of the audio recordings were prepared by a qualified transcription service. This transcription followed a simplified approach guided by the Dresing/Pehl rules, a method that involves a certain degree of data smoothing [33]. The quotations have been translated into English for the purpose of publication. Access to the transcripts was granted to all participants upon request. The interview material was analyzed pseudonymized using qualitative content analysis according to Kuckartz and Rädiker [34]. A categorization system was developed in the context of the overall formative evaluation to structure the data material in an iterative process, combining inductive and deductive categorization approaches. Most of the primary categories were derived based on the questions in the interview guide. The subsequent assignment of text passages to subcategories was conducted in a gradual manner. The MAXQDA software was used to structure and code the interview material [35].

To ensure intercoder reliability, a second independent scientific researcher, who was not involved in the DigiNet-project, participated in the process. The second coder independently coded the interview material over several rounds using the current categorization system. Subsequently, the concordances with regard to the categories were determined. Discrepancies were addressed during meetings and, where necessary, minor modifications to the categorization system were implemented.

As the implementation of PROs was analyzed as a primary focus of the process evaluation, they constituted one primary category in the categorization system. For the subsequent analysis and presentation of the interview data, the framework developed by Grol and Wensing was employed [36]. Based on review of the literature, they developed a multilevel approach: healthcare was divided into six dimensions, each of which had the capacity to exert an impact on the implementation process. These are: the innovation itself, the individual professional, the patient, the organizational context, the social context, and the economic and political context. Each dimension encompasses specific facilitators and barriers relevant to the implementation of PROs and allows for a structured assessment of multilevel influencing factors. Table 1 provides an overview of the dimensions with adapted definitions tailored to the context of PRO integration in personalized lung cancer care.

**Table 1:** Dimensions of healthcare by Grol and Wensing adapted for the analysis of PRO implementation [36]

Dimension	Description with reference to PROs in personalized lung cancer care
Innovation	Characteristics of the innovation itself, such as the practicality, usefulness, or adaptability of PROs as tools for symptom and quality of life monitoring. Relevance, clarity, and clinical applicability are central aspects.
Individual professional	Attributes and attitudes of healthcare professionals, including their acceptance of PROs, confidence in their usefulness, and digital or methodological competencies for using PRO tools.
Patient	Factors on the patient side, such as motivation, health literacy, digital skills, perceived benefit, and potential burden related to participation in PRO assessments.
Organizational context	Conditions within the healthcare organization, such as availability of resources, IT infrastructure, workflows, role clarity, and interprofessional collaboration in utilizing PROs in routine care.
Social context	Influences from the broader social and systemic environment, including peer attitudes, leadership support, institutional culture, and regulatory requirements concerning the use of PROs; and the social situation or support of patients.
Economic and political context	Funding, regulations, and policies related to PRO implementation in cancer care. Not covered by interview guide.

## Results

In round 1 (2023), *N* = 25 HCPs were interviewed, including *n* = 9 physicians and *n* = 16 assistants. In round 2 (2024), there were *N* = 26 HCPs, of which *n* = 8 were physicians and *n* = 18 were assistants. *N* = 11 HCPs participated in both rounds. This resulted in 51 interviews with 40 HCPs in total. The patient interviews included *N* = 24 patients in 2023 and *N* = 10 patients in 2024. The duration of the interviews with HCPs ranged from a minimum of 10 min to a maximum of 56 min, with a mean duration of 24 min. Interviews conducted with patients ranged from 5 to 64 min, with a mean duration of 22 min. Some patient interviews were brief, as responses were largely succinct and additional prompts did not generate further discussion. Characteristics of interviewed participants, including their gender, age, and study region, are presented in Table 2.

**Table 2:** Characteristics of interviewed HCPs and patients

	HCPs *N* = 40	Patients *N* = 34
**Gender, ** ***n*** **(%)**		
Female	25 (62.5)	14 (41.2)
Male	15 (37.5)	20 (58.8)
**Age in years**,** n (%)**		
≤ 40	20 (50.0)	0
> 40 ≤ 60	20 (50.0)	12 (35.3)
> 60 ≤ 80	0 (0.0)	17 (50.0)
> 80	0 (0.0)	2 (5.9)
n.a.	0 (0.0)	3 (8.8)
**Study region**,** n (%)**		
North Rhine-Westphalia	15 (37.5)	16 (47.1)
Saxony	12 (30.0)	7 (20.6)
Berlin	10 (25.0)	3 (8.8)
Thuringia	1 (2.5)	2 (5.9)
Bavaria	2 (5.0)	3 (8.8)
n.a.	0 (0.0)	3 (8.8)

**Fig. 2:**
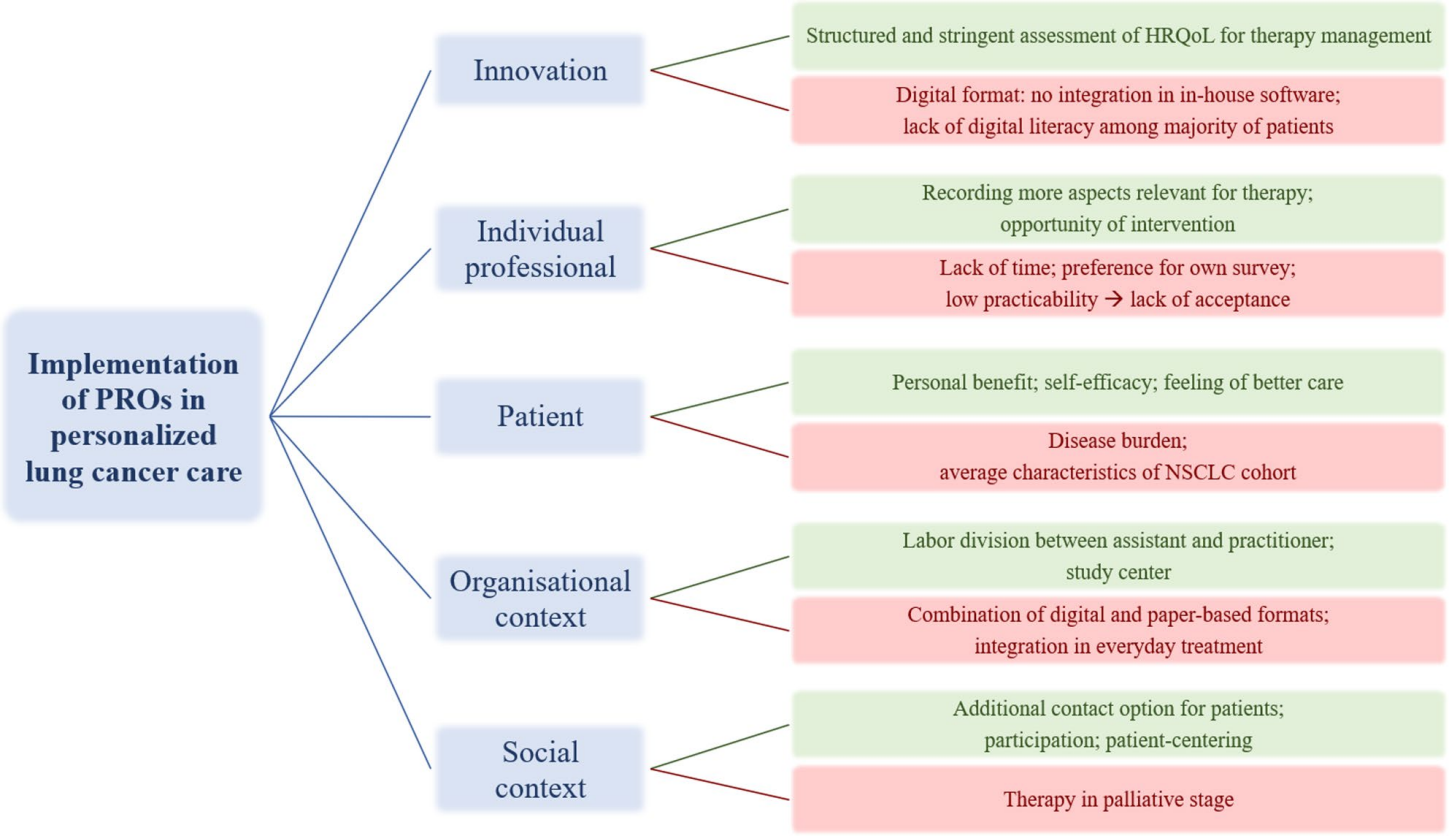
Barriers to and incentives for change at different levels of healthcare from Grol and Wensing [36]

Figure 2 provides an overview of the main influencing factors identified in this study and their assignment to the five domains of the Grol and Wensing framework.

### Innovation

#### Collection of PROs: digital via portal or paper-based

Of the 34 patients who participated in the interview, five did not complete any PROs. The choice between digital and paper-based formats was determined by the patients’ individual preferences. Among the 29 patients who regularly completed PROs, 20 reported using the patient portal. Most of them had already gained experience with digital media through professional or personal use and regarded the portal as highly user-friendly and intuitive.

It is quite simple, I would say, quite simple. It is very well structured and also, shall we say, logical in the order the questions are asked. (Patient (P) 5)

The digital data collection process was welcomed by certain individuals, specifically within the context of the digitalization of the healthcare sector, contingent on assurances of data privacy. As stated by several patients, the subjective appraisal of digital data collection is dependent on their digital proficiency, which may render it less applicable to other patients.

Yes, I understand that the world is moving fast and the importance of digitalization that everyone keeps going on about, but I think that the paper version should not and probably will not die out, because there will still be people like me, or also down to the fact that maybe - I don’t know the figures - but maybe it’s mostly for older people, who are not so familiar with these things. (P 25)

From the perspective of the HCPs, the need to log into an additional system in order to access the visualized PRO results was a factor hindering the integration of PROs into clinical decision-making and routine care. As such, integration with in-house software was recommended. In addition to this, it would be advantageous for the treating physician to receive a prompt, automated notification if the patient’s condition deteriorates.

Sometimes patients are doing well, sometimes not so much. If they are doing well, I don’t necessarily have to use it. But if they take a turn for the worse, that’s something I need to know. And I don’t get that feedback here –I have to actively look at the questionnaires, I have to look at all the questionnaires to get an overall impression of the patient […] and I don’t get just a quick summary. And no direct message, either: ‘Hang on, look, this patient is getting worse. Come and take a look.’ (HCP 36 & HCP 37)

Nine patients selected the paper-based format, with the majority citing their lack of prior experience with the internet and digital media as the primary reason for their decision. A number of individuals have expressed a reluctance to utilize the portal, stating concerns regarding the collection and dissemination of digital data, as well as a lack of trust in the principles of data collection and dissemination.

I mean, I’m sort of an in-between generation, you see? We do everything online when we have to, but we still prefer to have a piece of paper in our hand. (P 34)

I’m not against using that kind of portal. But […] any digital footprint or any digital trail that you leave behind – that can backfire on you eventually, and I don’t want that. (P 25)

The perspective of the HCPs has also revealed that a lack of digital proficiency in the DigiNet cohort is an impediment to the utilization of the portal. Assistants specifically highlighted the issue of patients who lacked access to an email address, necessitating the involvement of family members to facilitate registration on the patient portal. The participation and attrition rates were found to be comparable for both the digital and paper-based formats.

You have to understand what kind of people are taking part. Some of them are over 65, 70, some of them don’t have their own e-mail address; they get their children to help them. That’s just something you’ve got to bear in mind. We’re not doing this for 40-year-olds. The response would certainly be better if we were. (HCP 13)

#### Content, structure and assessment interval

The majority of patients found the questions to be comprehensible, succinct and not overly detailed. The subjective perception of whether this was adequate varied between individuals. One criticism leveled at the questions was that concomitant diseases and the emotional state of the patient, which is frequently significantly influenced by the disease burden, were not sufficiently taken into consideration. It was suggested that the inclusion of free text fields would facilitate the provision of additional information and the elaboration of aspects that have been evaluated using a categorical scale. This would enable the treating physician to interpret the patient’s individual situation in a more nuanced way. Participants’ perception of the appropriateness of the assessment interval was contingent upon the progression of the illness and the patient’s condition. The majority of them deemed a four-week interval to be appropriate.

In the medical field, especially when it comes to cancer treatment and cancer, there are also emotions involved. And you can’t just ask someone to answer those kinds of questions with a clear yes or no, 0 or 1. (P 2)

I would maybe add a free text line in some places […]. ‘Fair’ or ‘bad’ or ‘good’ are answers, but sometimes you want to add more specifics, and there aren’t many places you can do that. (P 6)

### Individual professional

#### Benefit

From the perspective of multiple physicians who incorporate PROs into the treatment of their patients, the utilization of PROs facilitated the discussion of topics that would not otherwise be addressed during physician consultations due to time constraints or the general distress experienced by patients during treatment appointments. If the treating physician had reviewed the results in advance and identified any notable deviations, these issues would be addressed with the patient in a targeted manner. In the event of a deterioration in symptoms or the general condition, an intervention can be considered.

Yes, I think that you can also see what kind of complaints the patients have. […] what the real problems are or what they are suffering from, whether there are any side effects, they don’t always tell us these things, and then we find out through the questionnaire. (HCP 23)

A further advantage is that relevant side effects are recorded in a more structured manner, thus facilitating a more precise stratification, particularly through the use of visualized outcome data.

I think so; I mean you also have a strict chronology, of course, that you can refer to again and again. It goes without saying that it enables much more precise documentation and better stratification than just five sentences that you write in your own document, which you don’t really read over because you can visualize it much better. (HCP 35)

A number of physicians have refrained from incorporating PROs into their treatment regimens due to a perceived imbalance between the additional effort and the anticipated benefits. This is attributable to the preference for collecting information through personal interviews or internally used questionnaires.

That’s not going to happen with us because, once again, we are a study team. We are separated from the clinical routine, and the clinical routine is so full that such a requirement cannot be met in our structure. (HCP 10)

This issue has been addressed in the course of the project by the implementation of a traffic light system to visualize the results, thereby facilitating the use of the color red to indicate significant alterations. This system was perceived as more intuitive to use; however, it did not result in greater integration of PROs.

I think the idea is good in principle, but for me, it doesn’t address the main problem we have with the structure of DigiNet. (HCP 11)

### Patient

#### Benefit

For the majority of patients who participate regularly, completion of PRO questionnaires was associated with personal benefit. The process of reflecting upon the questions assisted a number of individuals in becoming more conscious of their health status, thereby sharpening their perception of emerging adverse effects.

But dealing with the questions regularly means I keep regular track of my own state of health and that helps me to cope with the illness. […] When I can see that I have answered the questions the same way as last time, that there has been no deterioration, I am satisfied. […] It helps if you don’t just rely on your feelings, but if you think about it rationally: ‘Okay, it hasn’t really got any worse or anything,’ and that gives you courage for the next time. (P 1)

When there are no direct personal benefits and a lack of integration of PRO results into clinical consultations, many patients were motivated to participate in the PRO assessment out of a desire to support scientific research and contribute to the improvement of future patient care.

It can help not just me, but also the research for patients who will be treated further down the line. I’m not the only one making these statements – there must be lots of other people taking part in the study. (P 5)

From patients’ perspectives, the value of the PROs for their own treatment was considered to be rather small, primarily due to the perception that PROs were not meaningfully incorporated during medical consultations. Patients who reported that PROs offered them a personal benefit prefer a more proactive use of the results in the treatment process.

After all, going through all this would take up too much of my time during the consultation. What’s the point of the doctor looking at the form if it means they won’t have time to talk to me? (P 34)

Five of the interviewed participants reported that they had not completed any PROs. The underlying reason for this was typically the absence of information regarding the use of PROs as part of the study. One patient declined to participate in the PRO survey due to the emotional distress she was experiencing in the immediate aftermath of receiving the diagnosis. From the perspective of the HCPs, the use of PROs was deemed to be unfeasible in the context of the specific medical condition of stage IV NSCLC. The assistants responsible for direct patient contact expressed the view that the disease burden is frequently excessive in terms of both the patients’ emotional well-being and treatment-related adverse effects.

Because that is much too much for them in this situation. You have to understand, I mean, the patients have the disease, they get the treatment and they are happy to have even just a few days or hours where they feel relatively well and can forget about the disease. That doesn’t just apply to your project. As I said, 90% of the patients reject the idea of having to do homework, of having to think about their illness during that time. It’s all too stressful. (HCP 32)

#### Participation

It was estimated by HCPs that approximately 30% of DigiNet patients participated in the PRO survey. According to a research assistant, frequent reminders were necessary. Some patients discontinued the survey during the course of the study due to the perceived burden.

Put it this way: According to our observations, the patient factor remains in - how should I put it? - a few large groups called ‘no interest at all’, ‘interested, but overwhelmed’ or even ‘I don’t really want any more apps on my phone or tablet.’ (HCP 27)

Furthermore, sociodemographic characteristics of NSCLC patients, including age, appeared to influence willingness to participate. There have also been reports of issues related to social status and language barriers. In contrast, many HCPs perceived a higher level of engagement among patients who consistently complete PROs, suggesting a willingness to participate in the survey and an appreciation of its potential value.

And then there’s the other side, which I’ve come to experience more and more. I’ve met younger patients who have said: ‘Yes, we think it’s great that we have this networking, […] which is driving things forward.’ I also know patients who see a lot of opportunities and hope for networking and data collection and so on. (HCP 11)

### Organizational context

The organization of the PRO integration is typically determined by the specific conditions at each location. In many cases, a division of labor between the roles of assistant and treating physician was required. For instance, a study nurse may be responsible for helping patients access the survey, e.g. by registering them on the portal. If the timely review of the patients’ results by the treating physician was not possible, the assistant ensured that responses were reviewed regularly to identify any negative trends or signs of deterioration. If there was a need for intervention, the assistant would forward the responses to the treating physician for consideration.

I’m the one who goes through it all, and when I notice that a patient has ticked a box, especially on the scale that asks: ‘How are you feeling today?‘, and you compare that, and when you see that it’s going down and down over the past four weeks, I point that out and say: ‘Look at that and ask what’s going on,’ right? I think we tend to be the ones who pay a bit more attention to the bigger picture. (HCP 23)

The digital format has been demonstrated to exert a restrictive effect on the implementation process; a few HCPs have proposed modifications that aimed to promote the integration of PROs into the treatment routines to enhance their practicality. For instance, the utilization of tablets could increase the participation rate of patients by offering them the option to answer the questions with assistance from staff members, either during the waiting period or while receiving an infusion.

[…] and I think what would be a much better way to introduce something like that would be if either with apps or with a tablet you actually stopped the patients in the clinic or in the outpatient clinic to record these things there, maybe with a study nurse at the beginning, or something like that. (HCP 8)

In order to organize the paper-based format, the survey instruments were either dispatched to participants by post with a pre-paid return envelope, or administered during the one-site visit, in some cases outside the standard four-week interval. In select cases, paper questionnaires were administered to patients through interview techniques, either during in-person treatment or via telephone communication.

I basically did it the old-fashioned way, in the form of an interview, and that clicks with these patients from the 30s, 40s, 50s, 60s, more so than with the younger patients. I think that’s a way you could get more patients from these generations involved. (HCP 24)

Due to the considerable resources required, the combination of digital and paper-based formats was often regarded as impractical.

Well, I have to say that we have not really implemented the paper-based PROs, simply because it would have required much more staff. Our largest referring clinic said they didn’t have the resources to sit down with the patients and fill out PRO questionnaires. […] Yes, for paper-based surveys, I would say the biggest problem is just that they’re so resource-intensive. (HCP 7)

### Social context

In the context of direct communication, for instance when the assistant is responsible for the distribution of reminders or when the PRO results are discussed collectively with patients, an increased degree of interaction has been demonstrated to be beneficial and is highly regarded by patients.

I’ve also often conducted the interview by telephone. It helps you to build up a relationship with the patient, which is really great for the patients who are often alone at home and happy to receive a call. (HCP 9)

The engagement of patients in the examination of their own health status has been demonstrated to have a positive impact on their sense of self-efficacy, thereby facilitating their participation in the therapeutic process and enhancing their perception of receiving more comprehensive care. As outlined by several patients, PROs were regarded as beneficial in the preparation for upcoming medical consultations, particularly in terms of focusing on personally significant topics that are scheduled to be addressed during these appointments. Accordingly, PROs address the issue of patient-centeredness in medical consultations by means of an augmentation of the interaction between physician and patient.

I have actually asked questions in the doctor’s appointment that have arisen from the questions I have noticed […] and then I have actually gone into the doctor’s appointment with this question and asked: ’Could this be a side effect of the drug?’ (P 6)

HCPs with direct patient contact have questioned whether PROs are practical not only in terms of health status, but also in terms of the patients’ overall life situation, given that many patients with stage IV NSCLC are in a palliative situation.

It’s work, and for them it’s a real burden, because at some point they feel like they’re just patients and not people anymore. (HCP 33 & HCP 34)

### Economic and political context

Within the context of the economic and political dimensions, no significant facilitating or hindering factors to the implementation of PROs were identified. Firstly, these topics were not part of the interview guide. Secondly, most surveyed HCPs were not engaged in the financial activities of their institution.

## Discussion

This qualitative study constitutes a component of the formative evaluation of the innovation fund project DigiNet. The primary focus was the implementation of PROs as an intervention component within a national precision medicine program for lung cancer. The utilization of PROs was intended to enable healthcare professionals to more precisely manage personalized lung cancer care for their patients by means of a structured recording of their quality of life and symptoms.

The evaluation of the interviewed HCPs indicates that the incorporation of PROs into the existing treatment routine poses a considerable challenge in numerous clinical settings, with the practical challenges of implementation counteracting the potential benefits, such as prolongation of overall survival and improvement of HRQoL [12]. Consequently, the majority of the HCPs expressed a relatively low level of acceptance. Given the high workload and associated time pressure in daily clinical practice, the need to log into a separate system was identified as a significant barrier to the integration of PROs.

This finding is in accordance with the results of other studies on the implementation of PROs, which report an increase in the workload of all HCPs as a result of the incorporation of PROs [26, 28, 37–39].

The use of paper-based formats has been shown to have the advantageous effect of encouraging more patients to participate in the PRO surveys. This is particularly beneficial for those who are not proficient in digital applications or for whom personal reasons, such as concerns regarding data protection, render paper forms the preferred option [40]. From the perspective of HCPs, the combination of both formats, and also the utilization of the paper-based format in itself, is associated with a considerable increase in the need for resources. In order to generate a digital visualization of the paper-based questionnaires, it is necessary for the assistants to transfer the results into the central data platform. However, the availability of additional time and staff resources is a prerequisite for the overall organization of this, and one that is not met in many locations, be they university hospitals or medical practices.

From the patient’s perspective, it is evident that there are some perceived benefits to the intervention. These include the preparation for medical consultations and an increased level of mindfulness with regard to the patient’s health status. PROs can therefore be regarded as having a positive impact on patient participation and fostering patient-centered care. Further studies have been conducted in order to investigate the role of PROs in cancer care settings, with a view to examining the factors that promote or hinder this process. These studies have indicated a positive influence on the nature of conversations between patients and healthcare providers, which has evolved in a more open and in-depth direction [26, 38, 41].

Patients themselves are an important factor in implementation, as they are the ultimate beneficiaries of improved care. Nevertheless, their active participation is a prerequisite for this [42]. The cohort of NSCLC patients represents a significant impediment to this additional effort, primarily due to their comparatively low level of digital literacy, which is a prerequisite for utilizing the designated patient portal. This can be attributed to the mean age of 67 years for the entire DigiNet cohort. Furthermore, the physical and psychological burden resulting from the illness is such that the completion of survey instruments within the four-week interval is impracticable. In this regard, Govindaraj et al. posit that an undesirable consequence of regular PROs is that they act as a constant reminder of the illness [25].

Only limited conclusions can be derived concerning the precise reasons underlying the refusal of participation in the PRO survey. Some patients were not informed about this component of the intervention. Low acceptance of PRO use in treatment among HCPs may have affected patient participation. One participant cited the emotional distress experienced shortly after the initial diagnosis as the reason for their refusal. This may be a more widespread explanation for non-participation [43, 44].

The HCPs’ impression that only approximately one third of patients in DigiNet completed the questionnaires is a finding that is consistent with the data monitoring results, which show that the final participation rate is 36.3%. Given that the majority of patients are in a palliative situation, which frequently precludes the tolerance of additional stress, the utilization of PROs in lower NSCLC stages may be a more pragmatic approach. In accordance with the findings of other studies, in which PROs were also examined in a palliative care context, it was found that a high burden of disease is an obstacle to active participation in such an intervention [16, 45, 46]. From the perspective of various HCPs, the end-of-life setting is characterized as a delicate situation, which is often accompanied by a significant burden for the majority of individuals affected and is frequently marked by a progressive deterioration in health [16, 45]. This has been shown to have a negative impact on both the willingness and motivation of respondents to complete survey questionnaires regularly [16, 45, 46]. To address this obstacle, a careful consideration by the relevant personnel may be required, assessing the overall condition of a patient and determining their suitability for participation [37, 45]. Future iterations should adopt a selective-enrolment model that prioritizes (i) patients on systemic therapy with actionable alerts, (ii) individuals expressing high self-management motivation, and (iii) those with adequate informal support.

Overall, patient-related barriers to PRO participation appear to be strongly context-dependent, particularly regarding disease stage and individual capacities. These findings highlight the importance of flexible, tailored implementation strategies to ensure feasibility while maintaining meaningful patient involvement.

PROs in lung cancer therapy have the potential to enhance patient care by enabling health-care professionals to intervene on the basis of the results, and by allowing patients to derive a personal benefit from completing the assessments [47]. However, an analysis of both perspectives in the DigiNet project reveals that integrating them into the treatment routine is challenging due to insufficient resources and a generally low level of patient participation. These findings are consistent with previous studies reporting that the complex nature of PROs does not always align with established processes of routine care [16, 24, 39, 48, 49].

Importantly, many of the challenges identified relate to contextual and organizational factors rather than to PRO use per se. Addressing these barriers is therefore essential to support sustainable implementation and to realize the potential benefits of PROs in personalized lung cancer care [50].

### Strengths and limitations

A notable strength of this study is its integration within a broader research project, particularly in terms of its emphasis on a specific component within the framework of formative evaluation. Semi-structured interviews are a suitable method for obtaining an overview of the subjective perspectives of all the individuals involved in the implementation process. A prerequisite for the implementation of PROs is the acceptance of both perspectives in equal measure. The higher proportion of respondents who are assistants indicates that the viewpoint of personnel involved in the majority of the work processes that facilitate integration into treatment is considered.

The iterative process of coding was conducted by two researchers. A limitation inherent to the sampling method is that HCPs and patients who are more motivated to participate in studies and PROs on a regular basis are more likely to participate in interviews. A minor proportion of the interviewed patients did not participate in the PRO survey, thereby limiting the capacity to draw conclusions regarding the most prevalent reasons for refusal. This limitation was addressed by indirect explanations from HCPs. The multidisciplinary background of the research team and their prior experience in qualitative research may have influenced data collection and analytic interpretation. To reduce potential bias, interviews and coding decisions were discussed iteratively within the research group following the COREQ framework. Nevertheless, the influence of researcher presumptions – otherwise termed ‘research reflexivity’ – cannot be disregarded entirely.

An important aspect of this study is that it focuses on patients with advanced NSCLC, many of whom are in a palliative stage of care. This context represents a particularly sensitive phase of life in which regular PRO assessment may be demanding. At the same time, it highlights the value of PROs in making patient needs visible, supporting patient-centered care, and offering some patients personal benefits such as reflection and a sense of self-efficacy.

## Conclusions

While the findings of our qualitative study highlight several relevant barriers – particularly related to disease burden, digital literacy, workflow integration, and resource constraints – they also demonstrate that PRO use is perceived as valuable when it meaningfully informs consultations and supports patient-centered care.

The results indicate that PROs need to be carefully tailored to both the clinical and patient context. This includes flexible modes of completion that account for patients’ disease burden, as well as a higher level of practicability for HCPs in routine care. This objective can be achieved by integrating visualized results into electronic health records and clinical management systems which are used in routine care and by providing targeted notifications when a patient’s condition has deteriorated significantly, thus enabling timely intervention.

Based on our findings, future implementation efforts should focus on (1) aligning PRO collection with patients’ capacities and disease burden, (2) ensuring seamless technical and organizational integration into routine care, and (3) providing adequate support and resources for HCPs to enable the effective use of PRO data.

In conclusion, although the implementation of PROs in personalized lung cancer care is complex, this study indicates that many of the challenges identified are modifiable. By explicitly addressing key barriers and leveraging identified facilitators, PROs hold considerable potential to facilitate more structured symptom monitoring, enhance communication and support patient-centered care of advanced NSCLC.

## Data Availability

The data sets generated and/or analyzed as part of the current study cannot be disclosed due to the lack of permission from the patients. However, an overview of the main and subcategories based on the analysis of the transcribed interviews can be requested from the corresponding author upon justified request.

## Abbreviations

DigiNet: [project acronym]
HCPs: Healthcare providers
HRQoL: Health-related quality of life
NSCLC: Non-small-cell lung cancer
PROs: Patient-Reported Outcomes
